# Editorial: Advances in neuroimaging and its applications on biomedical devices

**DOI:** 10.3389/fnhum.2024.1374203

**Published:** 2024-02-22

**Authors:** Chen Li

**Affiliations:** Research Group for Microscopic Image and Medical Image Analysis, College of Medicine and Biological Information Engineering, Northeastern University, Shenyang, China

**Keywords:** brain imaging, data analysis, preclinical application, computer-assisted diagnosis, machine learning

Epilepsy is characterized by the sudden abnormal discharge of neurons in the brain, leading to temporary disruptions in brain function. The diagnosis of epilepsy is challenging for two main reasons. Firstly, various factors, including fever and certain medications, can cause symptoms similar to those of epilepsy. Secondly, capturing diagnostic electroencephalogram (EEG) signals for epilepsy in clinical practice is challenging. On the one hand, if a patient's epileptic seizures are infrequent, it is challenging to detect them at the right time. On the other hand, some types of epilepsy, primarily characterized by cognitive impairment, may not exhibit abnormal findings in the EEG. Neurology experts manually analyze EEG signals for epilepsy diagnosis. The non-stationarity and complexity of EEG signals make this task prone to errors, time-consuming, and expensive. Therefore, the development of automatic epilepsy detection technology is crucial to ensure the proper identification and treatment of this condition.

Wu et al. proposed an end-to-end epileptic seizure prediction method using Long Short-Term Memory networks (LSTM). The method achieved an average sensitivity of 91.76% and a false prediction rate (FPR) of 0.29/h on the Boston Children's Hospital-Massachusetts Institute of Technology (CHB-MIT) scalp electroencephalogram (EEG) database. By introducing the postictal stage as an additional category, the method's performance in epileptic seizure prediction further improved, achieving a higher sensitivity of 92.17% and a lower FPR of 0.27/h. The average warning time was 44.46 min. This has significant implications for clinical practitioners, enabling them to issue timely warnings for epileptic events in patients (Wu et al.).

Li A. et al. proposed a method involved screening for cognitive impairment using wearable devices and digital cognitive tests. Ren et al. demonstrated that brain network damage could lead to memory deficits in Alzheimer's disease (AD) and mild cognitive impairment (LMCI). Li M. et al. reviewed the progress of artificial intelligence applications in MRI for diagnosing primary headaches. Li X. et al. reviewed the application of machine learning in the treatment of Obsessive-Compulsive Disorder (OCD). Kaptan et al. reviewed the assessment of the human corticospinal cord using imaging techniques, highlighting recent developments and future avenues in this field.

The progress in hardware and software has driven the development of artificial intelligence in the field of medicine. Advancements in imaging methods and data processing techniques enable a more in-depth exploration of artificial intelligence applications in radiology, offering new and updated perspectives. However, significant variations in equipment and institutional protocols result in noticeable differences in images of the same modality obtained from different centers. Consequently, establishing targeted and more standardized imaging protocols for neuroimaging remains a challenging task that necessitates ongoing efforts from researchers.

## Author contributions

CL: Conceptualization, Funding acquisition, Writing—original draft, Writing—review & editing.

